# Supplementary material for: Sensory Preference and Professional Profile Affinity Definition of Endangered Native Breed Eggs Compared to Commercial Laying Lineages’ Eggs
Source: Animals (Basel). 2019 Nov 5;9(11):920. doi: 10.3390/ani9110920 (PMC6912648; doi:10.3390/ani9110920)
Supplement: Supplementary file 1 [file animals-09-00920-s001.zip › Supplementary Table S1.docx]

**Supplementary Table S1.** Recipe and chemical composition of the compound feed used for feeding the hen sets in the study.

| **Utrerana breed and Leghorn Lohmann LSL-Classic lineage fed on commercial conditions** | | **Leghorn Lohmann LSL-Classic lineage fed on ecological conditions^a^** | |
| --- | --- | --- | --- |
| *Recipe* | | *Recipe* | |
| Corn | | Corn | |
| Wheat | | Wheat | |
| Shelled toasted soybeans flour | | Shelled toasted soybeans flour | |
| Calcium carbonate | | Calcium carbonate | |
| Barley | | Barley | |
| Monocalcium phosphate | | Monocalcium phosphate | |
| Soybean oil | | Soybean oil | |
| Sodium chloride | | Sodium chloride | |
| Sodium bicarbonate | | Sodium bicarbonate | |
| *Chemical composition (%)* | | *Chemical composition (%)* | |
| Crude protein | 15.7 | Crude protein | 15.8 |
| Crude fat and oils | 3.6 | Crude fat and oils | 4.2 |
| Crude fiber | 2.4 | Crude fiber | 3.8 |
| Crude ashes | 14 | Crude ashes | 12.4 |
| Calcium | 4.1 | Calcium | 4.10 |
| Phosphorus | 0.66 | Phosphorus | 0.65 |
| Sodium | 0.15 | Sodium | 0.10 |
| Methionine | 0.38 | Methionine | 0.30 |
| Lysine | 0.79 | Lysine | 0.80 |
| ^a^The selection of the ecologic compound feed to be fed to the hens was chosen following the premises described in the Commission Regulation (EC) No 889/2008 of 5 September 2008 laying down detailed rules for the implementation of Council Regulation (EC) No 834/2007 on organic production and labelling of organic products with regard to organic production, labelling and control. | | | |
