# Supplementary material for: Sensory Preference and Professional Profile Affinity Definition of Endangered Native Breed Eggs Compared to Commercial Laying Lineages’ Eggs
Source: Animals (Basel). 2019 Nov 5;9(11):920. doi: 10.3390/ani9110920 (PMC6912648; doi:10.3390/ani9110920)
Supplement: Supplementary file 1 [file animals-09-00920-s001.zip › Supplementary Table S3.docx]

**Supplementary Table S3.** Scales for Utrerana native hen variables included in the sets of egg sensory attributes, Panelist diet habits, production context awareness, product consciousness, cuisine applicability and panelist characterization as perceived by cuisine instructed panelists (clustering set in bold).

| **Sensory attributes** (Yolk colour, white colour, smell, flavour, texture, overall value, whole egg visual value and broken egg visual value) | (1) I extremely dislike it |
| --- | --- |
|  | (2) I dislike it a lot |
|  | (3) I dislike it moderately |
|  | (4) I slightly dislike it |
|  | (5) I like it |
|  | (6) I slightly like it |
|  | (7) I like it moderately |
|  | (8) I like it a lot |
|  | (9) I extremely like it |
| **Sensory attributes** (White colour) | (1) I extremely dislike it to (8) I extremely like it |
| **Panelist diet habits** (Vegetable consumption,  fruit consumption, meat consumption  fish consumption and dairy consumption) | (1) No consumption |
|  | (2) One day/week |
|  | (3) 2 to 5 days/week |
|  | (4) 6 to 7 days/week |
| **Panelist diet habits** (Egg consumption) | (1) ≤2 eggs per week |
|  | (2) 2 to 4 eggs per week |
|  | (3) 4 to 6 eggs per week |
|  | (4) 6 to 10 eggs per week |
|  | (5) >10 eggs per week |
| **Panelist diet habits** (Ecological consumer) | (1) Yes |
|  | (2) No |
| **Production context awareness** (Free range hens, Drug prohibition, GMO banning), **product consciousness** (Product closeness, Endangered breed product, Seasonal product) and **cuisine applicability** (Egg in appetizers, Egg in pasta, Egg in soup, Egg in salad, Egg in main course) | (1) No importance to (10) Extremely important) |
| **Production context awareness** (Hen welfare and environment respect) and **cuisine applicability** (Egg in desserts) | (1) No importance to (8) Extremely important) |
| **Product consciousness** (Ecological product, Andalusian autochthonous breed product and product closeness) | (1) No importance to (9) Extremely important) |
| **Product consciousness** (Utrerana knowledge) | (1) Customer acquainted to the breed |
|  | (2) Customers not acquainted to the breed |
| **Product consciousness** (Prices for commercial, free range or ecological eggs) | from 1 to 13, 1 to 15 and 1 to 17, respectively. |
| **Panelist characterization** (Age) | (1) ≤20 years old |
|  | (2) from 21 to 30 years old |
|  | (3) from 31 to 40 years old |
|  | (4) from 41 to 50 years old |
|  | (5) ≥51 years old |
| **Panelist characterization** (Sex) | (1) Man |
|  | (2) Woman |
| **Panelist characterization** (Academic level) | (1) No studies |
|  | (2) Primary school |
|  | (3) High school |
|  | (4) College or University studies |
