# Supplementary material for: Sensory Preference and Professional Profile Affinity Definition of Endangered Native Breed Eggs Compared to Commercial Laying Lineages’ Eggs
Source: Animals (Basel). 2019 Nov 5;9(11):920. doi: 10.3390/ani9110920 (PMC6912648; doi:10.3390/ani9110920)
Supplement: Supplementary file 1 [file animals-09-00920-s001.zip › Supplementary Table S5.docx]

**Supplementary Table S5.** Kruskal Wallis H Ranks, Dunn's test and Bonferroni's significance correction and Median sorted by egg type for Utrerana native hen egg sensory attributes.

|  | Yolk colour | White colour | Smell | Flavour | Texture | Global value | Whole egg visual value | Broken egg visual value |
| --- | --- | --- | --- | --- | --- | --- | --- | --- |
| Chi-Square | 61.28 | 3.669 | 15.718 | 18.435 | 7.083 | 23.082 | 10.354 | 41.937 |
| df | 2 | 2 | 2 | 2 | 2 | 2 | 2 | 2 |
| Asymp. Significance | 0.000 | 0.160 | 0.000 | 0.000 | 0.029 | 0.000 | 0.006 | 0.000 |

|  | Egg Type pairwise comparison | Test Statistic | Sig. | Bonferroni Adj. Sig. |
| --- | --- | --- | --- | --- |
| Yolk colour | Commercial-Ecologic | 12.522 | 0.000 | 0.001 |
| Yolk colour | Commercial-Utrerana | 50.443 | 0.000 | 0.000 |
| Yolk colour | Ecologic-Utrerana | 20.666 | 0.000 | 0.000 |
| Smell | Commercial-Utrerana | 16.020 | 0.000 | 0.000 |
| Smell | Commercial-Ecologic | 2.002 | 0.157 | 0.471 |
| Smell | Utrerana-Ecologic | 2.776 | 0.096 | 0.287 |
| Flavour | Commercial-Ecologic | 7.880 | 0.005 | 0.015 |
| Flavour | Commercial-Utrerana | 13.333 | 0.000 | 0.001 |
| Flavour | Ecologic-Utrerana | 0.781 | 0.377 | 1.000 |
| Texture | Commercial-Utrerana | 1.222 | 0.269 | 0.807 |
| Texture | Commercial-Ecologic | 9.141 | 0.002 | 0.007 |
| Texture | Utrerana-Ecologic | 3.782 | 0.052 | 0.155 |
| Overall value | Commercial-Ecologic | -36.867 | 0.000 | 0.000 |
| Overall value | Commercial-Utrerana | -42.281 | 0.000 | 0.000 |
| Overall value | Ecologic-Utrerana | 5.414 | 0.572 | 1.000 |
| Whole egg visual value | Commercial-Ecologic | 11.782 | 0.001 | 0.002 |
| Whole egg visual value | Commercial-Utrerana | 21.014 | 0.000 | 0.000 |
| Whole egg visual value | Ecologic-Utrerana | 0.667 | 0.414 | 1.000 |
| Broken egg visual value | Commercial-Ecologic | 9.581 | 0.002 | 0.006 |
| Broken egg visual value | Commercial-Utrerana | 22.533 | 0.000 | 0.000 |
| Broken egg visual value | Ecologic-Utrerana | 3.125 | 0.077 | 0.231 |

| Egg type | Parameter | Yolk colour | White colour | Smell | Flavour | Texture | Overall value | Whole egg visual value | Broken egg visual value |
| --- | --- | --- | --- | --- | --- | --- | --- | --- | --- |
| Commercial | Median | 2 | 3 | 3 | 2 | 3 | 2 | 3 | 2 |
| Utrerana | Median | 5 | 3 | 4 | 4 | 3 | 4 | 3 | 4 |
| Ecologic | Median | 3 | 3 | 4 | 3 | 4 | 3 | 3 | 3 |
