# Supplementary material for: Sensory Preference and Professional Profile Affinity Definition of Endangered Native Breed Eggs Compared to Commercial Laying Lineages’ Eggs
Source: Animals (Basel). 2019 Nov 5;9(11):920. doi: 10.3390/ani9110920 (PMC6912648; doi:10.3390/ani9110920)
Supplement: Supplementary file 1 [file animals-09-00920-s001.zip › Supplementary Table S6.docx]

**Supplementary Table S6.** Components loadings for nonlinear canonical correlation analysis for Utrerana native hen egg sensory attributes (yellow), Panelist diet habits (red), production context awareness (purple), product consciousness (green), cuisine applicability (blue) and Panelist characterization (grey) as perceived by cuisine instructed panelists (n=192).

| Set | Variables | Dimension | |
| --- | --- | --- | --- |
|  |  | 1 | 2 |
| Egg sensory attributes | Yolk colour | -0.013 | -0.013 |
|  | White colour | 0.107 | -0.132 |
|  | Smell | 0.450 | -0.050 |
|  | Flavour | 0.097 | -0.167 |
|  | Texture | -0.146 | 0.028 |
|  | Overall value | 0.047 | -0.248 |
|  | Whole egg visual value | -0.617 | -0.167 |
|  | Broken egg visual value | 0.103 | 0.084 |
| Panelist diet habits | Egg consumption | 0.110 | -0.085 |
|  | Vegetable consumption | -0.042 | -0.351 |
|  | Fruit consumption | -0.199 | 0.155 |
|  | Meat consumption | 0.047 | -0.062 |
|  | Fish consumption | -0.930 | -0.246 |
|  | Dairy consumption | -0.139 | 0.173 |
|  | Number of eggs per week | 0.128 | -0.563 |
|  | Ecological consumer | -0.160 | 0.127 |
| Production context awareness | Hen welfare | 0.076 | -0.237 |
|  | Free range hens | -0.121 | -0.007 |
|  | Drug prohibition | 0.534 | 0.134 |
|  | Environment respect | 0.191 | -0.841 |
|  | GMO banning | -0.078 | -0.217 |
| Product consciousness | Product closeness | 0.192 | -0.829 |
|  | Utrerana knowledge | -0.055 | 0.090 |
|  | Commercial egg price | -0.839 | -0.257 |
|  | Free range egg price | -0.763 | -0.235 |
|  | Ecological egg price | -0.621 | -0.176 |
|  | Ecological product | 0.750 | 0.166 |
|  | Andalusian autochthonous breed product | 0.110 | -0.034 |
|  | Endangered breed product | 0.092 | -0.302 |
|  | Seasonal product | 0.043 | -0.019 |
| Cuisine applicability | Egg in desserts | 0.127 | -0.663 |
|  | Egg in appetizers | 0.123 | -0.010 |
|  | Egg in pasta | 0.195 | -0.069 |
|  | Egg in soup | 0.067 | 0.185 |
|  | Egg in salad | 0.579 | -0.109 |
|  | Egg in main course | 0.139 | -0.252 |
| Panelist characterization | Age | 0.154 | -0.168 |
|  | Sex | -0.140 | 0.026 |
|  | Academic level | 0.145 | -0.707 |
